# Making mobility-related disability better: a complex response to a complex problem

**DOI:** 10.1186/1741-7015-10-121

**Published:** 2012-10-15

**Authors:** Kenneth Rockwood

**Affiliations:** 1 Geriatric Medicine Research Unit, Dalhousie University and Queen Elizabeth Health Sciences Centre, 5955 Veterans' Memorial Lane, Halifax, NS B3H 2E1, Canada; 2 Division of Geriatric Medicine, Department of Medicine, Dalhousie University, Halifax, NS, Canada

**Keywords:** frailty, mobility, disability, multimorbidity, aged

## Abstract

Mobility disability in older adults can arise from single system problems, such as discrete musculoskeletal injury. In frail older adults, however, mobility disability is part of a complex web of problems. The approach to their rehabilitation must take that complexity into account, as is reported by Fairhall *et al. *First, their overall health state must be assessed, which is achieved by a comprehensive geriatric assessment. The assessment can show how a particular patient came to be disabled, so that an individualized care plan can be worked out. Whether this approach works in general can be evaluated by looking at group differences in mean mobility test scores. Knowing whether it has worked in the individual patient requires an individualized measure. This is because not every patient starts from the same point, and not every patient achieves success by aiming for the same goal. For one patient, walking unassisted for three metres would be a triumph; for another it would be a tragedy. Unless we understand the complexity of the needs of frail older adults, we will neither be able to treat them effectively nor evaluate our efforts sensibly.

Please see related article http://www.biomedcentral.com/1741-7015/10/120

## Background

As people become frailer, they typically move less well. At some early stage of frailty, mobility impairment can reflect a discrete problem, such as musculoskeletal injury or an isolated central or peripheral neurological problem. As frailty progresses, mobility and balance problems are universal [1]. By that stage, mobility disability no longer reflects discrete problems, - instead it represents multi-system impairments. In any older adult, multi-system impairments that give rise to mobility disability usually do not exist in isolation, but are seen in the presence of multiple, interacting medical and social problems that impair physiological reserve. In a word, the mobility impairment of frailty is typically complex.

Complex problems require complex responses. This is a special challenge in medicine. The triumph of scientific reductionism has made clear the merit of defining problems with great precision. The resulting history of increasing sub-specialization (especially when coupled with economic incentives) favours a narrow focus. Such a focus, however, is ill-suited to the problems of frail older adults, who are now amongst the chief consumers of health care. How are their problems, including seemingly discrete problems such as mobility disability, best managed?

## Discussion

In a recent article, Fairhall and colleagues from Sydney, Australia show how to sensibly address the complex problem of mobility-related disability in frail elderly people [2]. Their complex response begins with a comprehensive geriatric evaluation followed by a multicomponent and interdisciplinary intervention. The intervention was individualized, both in relation to the problem's cause and to the goals of participants and family members. This aspect of the intervention (a subset of a larger randomized clinical trial, as yet unpublished [3]) was rooted in 10, home-based, 45- to 60- minute physiotherapy sessions over 12 months. The sessions were front-end loaded, with five sessions scheduled in the first three months. They built on the Weight-bearing Exercise for Better Balance program [4]. Sensibly, the authors employed an evaluation strategy that embraced the complexity of their intervention. The Life Space Assessment assays disability outcomes at a societal level [5]. Whether the intervention met the individual goals of patients and caregivers was also evaluated.

Several points deserve highlighting. Individualized intervention is key, even when mobility disability reflects multisystem problems. However, not everyone will have the same path to it, or the same problems. To paraphrase Shaw, we must treat what patients have wrong with them and not just hope that they have wrong with them what we treat. To achieve this requires that we understand not just what is wrong with our patients, but what they need. One hears with amazement the complaints of many interventionist colleagues that they are too busy to know whether their patients are likely to achieve functional benefit from their interventions. This is troublesome. Patients typically undertake potentially risky procedures, not with the hope of having better X-rays or lab values, but with the hope of having better function. If we are not assessing risk and benefit in relation to what they expect, are we really getting informed consent? The comprehensive geriatric assessment (if and only if it gives rise to an individualized care plan) is a way for us to come to grips with the complexity of our patients' needs. Fairhall and colleagues show how this can be done.

Second, individualized care planning means that not everyone's intervention will be exactly the same; this in turn underlies the rationale for individualized outcome measurement. Here, individualization was achieved with a single mobility goal, but otherwise following the Goal Attainment Scaling method [6]. Some decry individualization is too subjective, but to understand clinical meaningfulness, we need the subject's impressions. Understanding how subjects feel about change insures relevance; the controlled design becomes the remedy for arbitrariness. Third, they extended their treatment to people with at least moderate cognitive impairment (a Mini-Mental State Examination (MMSE) [7] score as low as 18). Frailty and cognitive impairment commonly travel together [8] and while we must target our resources for maximal effectiveness, it is essential that we not adopt too refined an understanding of who we treat: no one should be too frail for a geriatrician.

## Future research

This work needs to be extended. The study is comparatively small (n = 241) and conducted at a single site. Generalizability is key, and should include other sites, rural areas and people with still lower MMSE scores. The authors' report of no impact on satisfaction with the ability to get out of the house is curious and requires follow-up. Although responsiveness had been demonstrated [9], measurement insensitivity seems possible, as the yes/no response to being asked "Do you get out of the house as much as you would like?" would not take into account changing expectations, or competing factors outside the intervention. Allowing a fuller response might also provide hints on how to make the intervention better.

I also am skeptical about defining frailty as a syndrome. Although inherent to the very widely used Cardiovascular Health Study (CHS) approach [10], and endorsed by leading commentators [11], it is not without its deficiencies [12,13], including infeasibility in very old adults [14]. Even so, viewing frailty narrowly as a syndrome and not a state ill serves clinical recognition of who is at greatest risk. This is because not everyone at increased risk is identified as frail, and because not everyone with the same CHS grade of frailty has the same level of risk. Perhaps the failure to recognize frailty as an at-risk state that arises from the complexity of deficit accumulation is why so much unnecessary effort and conceptual confusion now goes into debating the idea of multi-morbidity (a concept at once too narrow and too broad [15,16]). We need to get clear on this if we are to offer physicians the basic skill sets that they need to manage the complexity of frailty [13,17].

## Conclusions

The controlled trial of a multicomponent, interdiscliplinary approach to mobility-related disability of Fairhall and colleagues study has the ring of truth to it. It set achievable goals following a systemic assessment, and found that these goals could be met, with both subjective and objective improvement. For these reasons, the low uptake of home physiotherapy is a weakness. Even so, this low uptake should increase over time. Feasibility issues are common with any new program, and growing team confidence often results in greater patient uptake. Likewise, that one patient in seven could not set a goal, or that one in three of the goals became inappropriate due to deteriorating health, suggests that there is work to be done on this aspect of care planning.

Fairhall and colleagues have produced a useful study on an important and timely topic. It builds on a firm foundation, including the Frailty and Injuries: Cooperative Studies of Intervention Techniques trials [18], and joins other contemporary work, including that from Australia [19]. In having shown how mobility disability in frailty can be addressed, they further lay the foundation for comprehensive approaches to making life better for people who are frail.

## Abbreviations

CHS: Cardiovascular Health Study; MMSE: Mini-Mental State Examination.

## Competing interests

With his colleagues, KR has applied for funding to commercialize a version of the frailty index that views frailty as an at-risk health state and not as a specific syndrome or phenotype.

## Pre-publication history

The pre-publication history for this paper can be accessed here:

http://www.biomedcentral.com/1741-7015/10/121/prepub
